# A Blind Spot in Research on Foreign Language Effects in Judgment and Decision-Making

**DOI:** 10.3389/fpsyg.2018.00227

**Published:** 2018-03-13

**Authors:** Andrea Polonioli

**Affiliations:** Department of Philosophy, University of Birmingham, Birmingham, United Kingdom

**Keywords:** foreign language, decision-making, uncertainty, risk, heuristics, biases, rationality, emotions

## Abstract

One of the most fascinating topics of current investigation in the literature on judgment and decision-making concerns the exploration of foreign language effects (henceforth, FLE). Specifically, recent research suggests that presenting information in a foreign language helps reasoners make better choices. However, this piece aims at making scholars aware of a blind spot in this stream of research. In particular, research on FLE has imported only one view of judgment and decision-making, in which the heuristics that people use are seen as conducive to biases and, in turn, to costly mistakes. But heuristics are not necessarily a liability, and this article indicates two routes to push forward research on FLE in judgment and decision-making. First, research on FLE should be expanded to explore also classes of fast and frugal heuristics, which have been shown to lead to accurate predictions in several contexts characterized by uncertainty. Second, research on FLE should be open to challenge the interpretations given to previous FLE findings, since alternative accounts are plausible and not ruled out by evidence.

## Foreign Language Effects in Judgment and Decision-Making

Foreign language effects (FLE) have attracted a great deal of attention within research in the psychology of judgment and decision-making (Keysar et al., 2012; Costa et al., 2014a,b; Gao et al., 2015; Hadjichristidis et al., 2015; Hayakawa et al., 2016). It has been argued, for instance, that people thinking in a foreign language are more likely to display utilitarian behavior in moral dilemmas (Costa et al., 2014a; Geipel et al., 2015; Cipolletti et al., 2016). This article is concerned with the claim that thinking in a foreign tongue helps reasoners make better choices by reducing cognitive biases (Keysar et al., 2012; Costa et al., 2014a,b; Hadjichristidis et al., 2015; Hayakawa et al., 2016), which has been presented as having far-reaching implications. For instance, consider the literature on “nudges,” which comprises a number of interventions that policy makers as well as others with responsibilities over a group of individuals may implement to improve people’s decisions about health, wealth and happiness (Thaler and Sunstein, 2008). Notably, it has been recently claimed that “language could be used as a nudge to promote better choices" (Costa et al., 2017, p. 149).

Most of the work in the growing literature on FLE has interpreted these findings as emerging from a reduction in emotional processing. Specifically, thinking in a second language is supposed to elicit less intense emotional reactions compared to speaking in a first language. If the foreign language is learned in a classroom context, it is likely that the emotional connotation tied to the specific lexical items is not as rich as that of the lexical items of the native language, which are used in daily life interactions with relatives and friends (Costa et al., 2014a). But alternative accounts are also on offer, and a task for future research on FLE is to shed light on the underlying mechanisms responsible for the reported effects. In particular, foreign-language use might affect decisions by encouraging deliberative thinking rather than by decreasing emotional reactions (Hayakawa et al., 2017).

This paper maintains that whilst research on FLE has achieved important results, it can still be pushed forward by drawing attention to a set of overlooked issues from the judgment and decision-making literature. In particular, research on FLE has been clearly inspired by the view that the heuristics that people use are conducive to biases and, in turn, to costly mistakes. This view is, however, far from uncontroversial. Different perspectives on human judgment and decision-making have been offered (for surveys see Newell et al., 2007; Polonioli, 2014). In particular, the ecological rationality program of Gigerenzer et al. (2011) articulates how heuristics can be an asset, by characterizing these as adaptive.

The present paper suggests new avenues for future research on FLEs. More precisely, after a brief characterization of FLE research in Section “Judgment and Decision-Making under Risk and Uncertainty,” Section “Fast and Frugal Heuristics and FLE” explains that research on FLE in judgment and decision-making could be usefully expanded by exploring also classes of fast-and-frugal heuristics. Section “Reinterpreting Biases within FLE Research” explains instead that research on FLE should be open to reassess the interpretations given to previous FLE findings where alternative accounts are plausible and not ruled out by evidence. In underscoring these points, this article makes a case for greater conceptual as well as empirical work on FLE in judgment and decision-making.

## Judgment and Decision-Making Under Risk and Uncertainty

One key result of FLE research is that using a foreign language affects the contributions of intuition and deliberation in our decision-making. In particular, two recent studies report a reduction of the framing effect when participants make decisions in a foreign language (Keysar et al., 2012; Costa et al., 2014a). In the popular Asian Disease paradigm, people allegedly appear risk seeking when a situation is framed in terms of losses (e.g., 400 out of 600 people will die), and instead risk averse in those cases in which it is framed as gains (e.g., 200 out of 600 people will be saved). Yet this discrepancy tends to diminish when using a foreign language (Keysar et al., 2012; Costa et al., 2014a). Keysar et al. (2012) as well as Costa et al. (2014a) interpret their findings of a reduced framing effect in terms of an “emotional distance” account (i.e., a purportedly reduced emotionality in a foreign language setting). After all, framing effects have often been taken to arise when fast and emotion-driven processes compete with slow, deliberate, and rational processes (De Martino et al., 2006). Needless to say, there seem to be important implications for applied research. For instance, in medical contexts alternative options might be presented differently and patients’ vulnerability to framing effects seems to be of key importance.

Further studies have been offered to support the existence 176 of FLE effects in judgment and decision-making. Specifically, foreign language use has been shown to affect people’s judgments of risks and benefits by reducing the perception of risk and increasing the perception of benefit (Hadjichristidis et al., 2015). Moreover, research on FLE has focused on the hot-hand fallacy, namely the tendency to expect a positive outcome after a series of prior positive outcomes, even when the events are independent: it has been shown that using a foreign language might reduce this fallacy (Gao et al., 2015).

## Fast and Frugal Heuristics and Fle

Foreign language effects researchers have recently encouraged further work to explore the impact of using a foreign language on various other heuristics and biases. More precisely, it has been claimed that the effect of language should be studied also “on certain emotionally neutral biases such as *anchoring*, *hindsight bias*, and the *conjunction fallacy*” (Costa et al., 2017, p. 148). All of the abovementioned effects come, however, from the so-called heuristics-and-biases paradigm associated with the work of Daniel Kahneman and Amos Tversky which in the 1970s revolutionized research on human judgment and decision-making. Yet the wholesale adoption of this paradigm and framework by FLE researchers is by no means uncontroversial. In fact, there are reasons to think that a promising way to push forward research on FLE is by considering also another class of heuristics and effects that have been reported in research on judgment and decision-making. In particular, Gigerenzer and Todd (1999) have shown that an important class of so-called fast-and-frugal heuristics can also produce unbiased judgments that are at times even more accurate than those resulting from more complex cognitive strategies.

There are three main sets of considerations that seem to justify paying a good deal of attention to fast-and-frugal heuristics when addressing FLE. First, such strategies have been presented in the literature as key tools to deal with uncertainty. This is important because, as of yet, research on FLE has mostly focused on a particular class of decision-making contexts, i.e., those characterized by risk rather than uncertainty. But exploring fast-and-frugal heuristics in contexts characterized by uncertainty is expected to deliver a more comprehensive picture of FLE on thinking and decision-making. After all, the contexts of calculated risks that have attracted the attention of FLE researchers differ from many complex contexts characterized by uncertainty that we encounter in many real-life cases. Here, risk refers to situations of perfect knowledge, where the decision maker knows the probabilities of all outcomes for alternatives. Uncertainty refers instead to cases in which the probabilities cannot be expressed with mathematical precision. When comparing real-world situations and their uncertainty with the structure of calculated risk, it becomes evident that a huge deal of decision-making situations are of the former type (see, e.g., Volz and Gigerenzer, 2012; Mousavi and Gigerenzer, 2014). What research on fast-and-frugal heuristics has made clear is that investigating decision-making under uncertainty does not require introducing the complexity of a large world into the laboratory. It only requires investigating tasks in which not all alternatives, consequences, and probabilities are known for sure or presented by the researchers. More precisely, according to scholars in the framework of ecological rationality, individuals are provided with an adaptive toolbox, i.e., a set of heuristics that use only limited information and yet allow cognizers to successfully navigate the world of uncertainty (Gigerenzer and Selten, 2001; but see also Söllner and Bröder, 2016). Consider, for example, a simple heuristic that people seem to use in making inferences about an uncertain world (Goldstein and Gigerenzer, 2002):

*Recognition heuristic*: If one of two objects is recognized and the other is not, then infer that the recognized object has the higher value with respect to the criterion.

But another recognition-based heuristic is the *fluency heuristic*. This heuristic exploits our recognition memory and is defined this way: If two objects are recognized, and one of objects is retrieved more fluently, then do infer that the object has the higher value with respect to the criterion, where retrieval fluency is defined as how long it takes to retrieve a trace from long-term memory (see Schooler and Hertwig, 2005). These are obviously just few of the “simple heuristics” that could be explored, and in the class of one-good-reason heuristics it is also possible to find *take-the-best*, which is a strategy in which cues are ordered lexicographically, i.e., by comparing the cues one after the other, and using the first cue that discriminates as the one reason to yield the final decision (Gigerenzer and Goldstein, 1996). Such fast-and-frugal heuristics have been explored in a number of contexts. For example, consider the problem of predicting the purchasing behavior of customers. Wübben and von Wangenheim (2008) reported that, in the airline and apparel businesses, experienced managers use a simple heuristic, i.e., the hiatus heuristic: ‘Call *t* the number of months since a customer’s last purchase. Classify the customer as active if and only if *t* < 9.’ Importantly, exploring whether thinking in a foreign language affects also people’s use and selection of fast-and-frugal heuristics would provide us with a more complete picture of the effect of foreign languages on thinking and decision-making. More precisely, although it is still unclear to what extent people rely on these heuristics, they have been shown to capture interesting and important aspects of people’s decision-making.

Second, exploring this further class of heuristics is also likely to shed light on the boundaries of FLE. Does the fact that people are using a foreign language have an effect on the amount of information considered by decision-makers? This is an important question to address also in light of the fact that it is still unclear whether and to what extent reduced emotional resonance can account for FLE and fast-and-frugal heuristics are typically described as emotionally neutral. Establishing the boundaries of the phenomena has not proven easy so far. Consider for instance evidence about the impact of using a foreign language on performance on the cognitive reflection test (CRT), namely a test that includes logical problems that do not carry emotional connotations (Costa et al., 2014b). It was originally assumed in the literature that thinking in a foreign language would reduce only biases with an emotional basis and hence fail to improve performance on CRT (Keysar et al., 2012; Costa et al., 2014a). Yet in a previous study it was also suggested that ‘among [*their*] participants, those using a foreign language actually *outperformed* those using a native language in logical reasoning, with 60 and 47% of participants providing at least one correct answer out of three, respectively’ (Costa et al., 2014a, p. 5). This antecedent illustrates the complexity and yet importance of establishing the generalizability and boundaries of FLE.

Third, whilst heuristics in the heuristics-and-biases framework have typically been taken to be conducive to biases and costly mistakes, fast-and-frugal heuristics have also been associated with accurate predictions in a number of contexts (Gigerenzer and Todd, 1999). For instance, whilst the take-the-best heuristic (Gigerenzer and Goldstein, 1996) might violate transitivity and consider only limited information, the strategy has been shown to outperform regression in terms of predictive accuracy in a number of contexts. This suggests that, should the use of a foreign language in decision-making lead to reduced heuristic processing, it would still be unclear whether that also entailed decreased accuracy. Hence, whilst it is important to explore whether FLEs extend to fast-and-frugal heuristics, it is also interesting to assess the empirical accuracy of such heuristics. Further, since heuristics such as take-the-best or recognition can also be easily assessed in terms of their accuracy, this is another reason to explore such decision strategies and contexts in further research on FLE.

## Reinterpreting Biases Within Fle Research

Research on FLE should also be pushed forward by challenging previous interpretations of FLE findings. More precisely, it is also unclear whether those biases and effects that have been taken to be signs of irrationality in the literature on FLE should really be seen as such. Scholars interested in FLE should thus avoid taking claims about people’s tendency to bias and irrationality at face value. Debates over human rationality have represented an important chapter in the history of cognitive science (Stich, 1990; Gigerenzer, 1996; Kahneman and Tversky, 1996; Stein, 1996; Hammond, 2007; Bortolotti, 2011, 2014; Elqayam and Evans, 2011; Stanovich, 2011; Polonioli, 2014, 2015, 2016; Arkes et al., 2016). Notably, the claims that people are prone to biases and that these effects reveal irrationality are in fact controversial in the literature (e.g., Dawes and Mulford, 1996; Hertwig and Gigerenzer, 1999; Benoît and Dubra, 2011; Harris and Hahn, 2011; Polonioli, 2012) and a number of methodological objections have been offered to resist conclusions drawn within research on cognitive biases.

Interestingly, some of these concerns have been flagged with regard to findings purportedly revealing framing effects (Schick, 1991; Sher and McKenzie, 2006, 2008; Bermúdez, 2009, p. 90; Mandel, 2014; Tombu and Mandel, 2015), which have been discussed above and which have already attracted a great deal of attention in research on FLE on judgment and decision-making. In particular, as Sher and McKenzie (2008, p. 83) put it, “the normative analysis of framing effects cannot be neatly separated from the phenomena of *pragmatics*.” It seems sensible to take linguistic factors into account because virtually all framing tasks involve verbal descriptions of acts, outcomes or contingencies, all of which must be read and interpreted by the decision-makers reading such descriptions. For instance, in the context of the Asian disease problem, Mandel (2014) showed that people do not typically treat quantifiers as exact values. They tend to interpret “200 people will be saved” as meaning that at least 200 people will be saved (and maybe more) and “400 people will die” as meaning that “at least 400 people will die.” In such case, it is rational to prefer the risky choice in the die frame and the safe choice in the save frame: saving at least 200 lives out of 600 does not seem to be equivalent to letting at least 400 die. Moreover, when the sure option is fully described (200 lives will be saved and 400 lives won’t be saved), but the uncertain option is partially described (one-third probability of saving 600 in the positive frame versus two-thirds chance of 600 dying in the negative frame), the effect of frame was opposite to that observed with the standardly worded Asian Disease Problem. Importantly, as Mandel put it, “the linguistic implications of how numeric quantifiers are interpreted revealed in the present research extend to other types of framing, including attribute and health message framing, which, like risky-choice framing, often involve numeric quantification in language” (Mandel, 2014, p. 1194). Here, one testable hypothesis would be that the attenuation or elimination of framing effects in foreign language studies has to do with systematic differences in the interpretation of the numerical quantifiers. For instance, one possibility is that when processing tasks such as the Asian Disease Problem in a foreign language readers are less attuned to pragmatic factors that might be processed by natural language readers. Scholars seeking to test FLE should also consider scenarios in which the sure option has been fully described whereas the uncertain option has been partially described.

This shows that FLE interpretations of framing effects need to be critically reassessed, but these are by no means the only controversial instances of biases from the heuristics-and-biases framework and discussed by FLE scholars. In fact, whilst FLE researchers have encouraged greater work to explore the impact of speaking a foreign language on effects such as the conjunction fallacy, a number of studies have taken issues with widespread interpretations of this fallacy (cf. Gigerenzer, 2007), often appealing to pragmatic and linguistic factors. The classical finding is well-known: given the story of Linda, a person who took part in antinuclear demonstrations, majored in Philosophy, and some other activities, people judge of that person that it is more probable that she should be a bank teller and active in the feminist movement, than it is that she should be a bank teller. This phenomenon is usually interpreted as an indication of irrationality, because it violates the conjunction rule of probability theory, which states that the probability of a conjunction is always smaller than or equal to the probability of one of its conjuncts. Yet, it has been suggested that the phenomenon may represent a verbal misunderstanding of the probability concept: the prevailing statistical interpretation of probability (as relative frequency) does not appear to apply to colloquial language because everyday experience is seldom based on semantic frequency counts. Instead, the usual interpretation of “probability” may come close to such subjective criteria as “believability,” “degree of confidence,” “imaginability,” or “plausibility” (Fiedler, 1988, pp. 123–124). There is a huge literature on these concerns (e.g., Politzer and Noveck, 1991; Schwarz, 1994; Politzer, 2004; Moro, 2009) and it is important that FLE researchers carefully consider alternative explanations, considering whether dropping the word probability would impact the results, and asking people on which statement/option they would bet on, could they choose money by betting on an event.

The point that this section sought to drive home is that the uncritical adoption of one particular and not uncontroversial view of human rationality and decision-making within FLE research has been problematic. Not only FLE researchers need to focus on families of fast-and-frugal heuristics as well, but they also need to carefully consider reasoners’ goals and linguistic norms and phenomena that might give rise to alternative and yet plausible explanations for several biases explored within FLE research.

## Author Contributions

AP conceived of and wrote the paper.

## Conflict of Interest Statement

The author declares that the research was conducted in the absence of any commercial or financial relationships that could be construed as a potential conflict of interest.
